# Patterns and outcome of acute poisoning among children in rural Sri Lanka

**DOI:** 10.1186/s12887-018-1246-0

**Published:** 2018-08-18

**Authors:** M. B. K. C. Dayasiri, S. F. Jayamanne, C. Y. Jayasinghe

**Affiliations:** 1 Paediatrics, University Pediatrics unit, Lady Ridgeway Hospital for Children, Colombo, Sri Lanka; 2 Clinical Medicine, Faculty of Medicine, University of Kelaniya, Kelaniya, Sri Lanka; 3 Paediatics, Faculty of Medicine, University of Kelaniya, Kelaniya, Sri Lanka

**Keywords:** Poisoning, Children, Rural Sri Lanka

## Abstract

**Background:**

The burden of poisoning among children is largely underexplored in rural Sri Lanka. This study describes the patterns of demographic characteristics, poison related factors, clinical management and outcome following acute poisoning among children (9 months- 12 years) in rural Sri Lanka.

**Method:**

This hospital based multi-center study included Anuradhapura Teaching hospital, Polonnaruwa District General hospital, and 34 regional hospitals within Regional Director of Health Services in North Central province of Sri Lanka. The study assessed clinical profiles, poison related factors, clinical management, complications, harmful first aid practices, reasons for delayed management, complications and outcomes following acute poisoning over 7 years.

**Results:**

Among 1621 children with acute poisoning, the majority were in preschool age group. Household chemicals were accountable for 489 acute poisonings (30.2%). The most common poison was kerosene oil, followed by paracetamol. Most events occurred within their own domestic premises. Potentially harmful first aid measures were practiced by approximately one third of care givers. Reasons for delayed presentation at emergency center included lack of concern by family members regarding the urgency of the situation and lack of knowledge regarding possible complications. Complications were observed in 12.5% and the most common complication was chemical pneumonitis.

**Conclusions:**

Children with acute poisoing in rural Sri Lanka were predominantly preschoolers. They are poisonined mostly within their own housing premises. Kerosene oil, in addition to being the most common poison, had additional risks of aspiration pneumonia following potentially hazadrous first aid measures practised by the care givers. Complications though rare were potentially preventable by community education and awareness on timely attention to seek medical care, and avoidance of harmful first aid practices.

**Electronic supplementary material:**

The online version of this article (10.1186/s12887-018-1246-0) contains supplementary material, which is available to authorized users.

## Background

Poisoning is an important mode of accidental injury in the paediatric age group which is associated with significant mortality and morbidity. Poisoning and suspected poisoning in childhood are common and represent over half the cases referred to poison information services in some countries [1]. Over 345,000 people of all ages died worldwide as a result of accidental poisoning in 2004 according to the WHO Global Burden of Disease project and approximately 45,000 had been children and young people less than 20 years [2]. Poisoning was the fourth leading cause for accident related mortality among children [3] and the mortality rate was 1.8 per 10^5^ population [2]. Low and middle income countries observed four-fold higher mortality rate compared to high income countries [2]. Patterns of poisoning vary in different geographic areas globally with different socio-cultural and environmental risk factors. Despite having a significant impact on child health, global data on poisoning related morbidity are largely unavailable and regional data are not comparable due to variable access to healthcare services [2].

Medical management of poisoning in the paediatric age group is substantially costly. In the United States, the lifetime cost of poisonings to children was almost 400 million USD whilst medical treatment accounted for 9% [4]. Total expenditure for medical treatment of poisonings in the United States was estimated at $3 billion a year whilst an average of $925 spent per case [5]. Acute poisoning is an important clinical problem in Sri Lanka which has a substantial economic impact on the health service of the country. A Sri Lankan study reported that cost of treatment of patients following oleander poisoning with and without antitoxin as US$ 691.6 and US$ 58.6 per patient, respectively [6]. Another study from rural Sri Lanka revealed that ward staff input and medications had the highest expenditure whilst an average US$ 31.83 was spent on each adult patient with poisoning [7]. The average cost of transferring was US$ 14.03 per patient [7]. Approximate total government cost of treating all poisonings in Sri Lanka for the year 2004 was US$ 866,304 [7]. The data on economic loss and financial costs of managing children with poisoning in Sri Lanka are currently unavailable. It has been revealed that for every dollar spent on poison prevention services, an estimated $7 can be saved for medical expenditure by reducing the number of medically treated poisonings [5]. It is therefore vital that poison prevention authorities are well informed regarding patterns of poisoning among children to plan effective preventive strategies.

There are only a few studies published based on the patterns of acute poisoning among children in Sri Lanka. Lucas et al. [8] studied patterns of acute poisoning among children over 15 years (1985–2000) at the largest children’s hospital in Colombo, Sri Lanka. The study was based predominantly on an urban population. Fernando et al. evaluated patterns of acute poisoning in predominantly urban Western province among children who were below 15 years in 1986 and reported kerosene oil as the most common poison. Case fatality rate was 3.2% [9]. Childhood poisoning trends have changed over the years with socio-cultural development and improved living standards and there are no studies published in Sri Lanka for more than two decades. Further, there are no detailed paediatric studies on rural populations of Sri Lanka to date. Availability of such information would indefinitely benefit poison management centers in planning preventive interventions, educating the community and allocating scarce resources more efficiently. The purpose of the current study was to evaluate all pediatric patients admitted to the two major hospitals and 34 regional hospitals within RDHS (Regional Director of Health Services) in the predominantly rural North Central province of Sri Lanka for a period of 7 years. All children with a clinical diagnosis of acute poisoning were studied with a view to determine the patterns of clinical profiles, poison related factors, harmful first aid measures, clinical management, complications, and outcome of acute paediatric poisoning.

## Methods

### Study population and setting

This multi-center observational study was hospital based and conducted in the North-Central province (NCP) of Sri Lanka which provides dwelling to a predominantly rural population. The province accommodates a population of 1,259,567 and 30.2% are agricultural workers [10]. The study included the two major hospitals of the province which function as referral centers for the entire province and they were Anuradhapura Teaching hospital and Polonnaruwa District General hospital. Data were also collected from 34 other regional hospitals which function under RDHS of the North Central province.

### Participants

This study involved all in-patient children who presented with either acute unintentional or intentional poisoning. Children were recruited to the observational study after their poisoning events were confirmed following the initial evaluation at the hospital emergency department and subsequently at general paediatric wards. All children who were between 9 months to 12 years of age were recruited to the study. Food poisoning, snake envenomation, allergic reactions and adverse drug reactions which can be considered in the purview of toxicology were omitted in the study. Children with doubtful poisoning where there was no clear aetiology following thorough evaluation by the principal investigator were also excluded from the study.

### Study design

Data collections in the study were carried out in both prospective and retrospective directions and the time period covered by the study was 7 years (2007 February – 2014 January). The study was conducted in four major arms- (1) A 2 year prospective observational study (2012 February-2014 January) at Anuradhapura Teaching hospital (ATH), (2) A 2 year prospective observational study (2012 February- 2014 January) at Polonnaruwa District General hospital (PDGH), (3) 1 year prospective observational study at 34 regional hospitals within RDHS of NCP (2013 January- 2014 January), (4) A 5 year retrospective observational study at Anuradhapura Teaching hospital (2007 February- 2012 January). The methodology used in the current study has been described in a previous study by the same authors on pesticide poisoning among children [11].

### Data collection

Data were collected from the caregivers of children in all three prospective observational studies. Mothers were interviewed in most encounters and fathers or other caregivers were interviewed only when mothers were not available to participate in the study. Major part of the data collection was conducted at Anuradhapura Teaching hospital and data collection from all caregivers in the prospective study in that setting was done by the principal investigator himself to minimize interviewer bias. Interviews with the caregivers were conducted on the same day of admission to minimize possible recall bias. Data were collected using a pretested multi-structured questionnaire (Additional file 1) which comprised of questions to identify demographic data, type and circumstances of poisoning, poison related factors, location of poisoning, first aid measures, clinical management, reasons for delayed management, complications, and outcome following acute poisoning. Complications were defined for the study as acute hepatic injury (rise of alanine transaminase > 180 U/L, cardiac arrhythmias (all types of pathological cardiac arrhythmias including sinus bradycardia) and chemical pneumonitis (clinical and radiological evidence of pneumonia) whilst aspiration was determined based on medical history. The questionnaire was pretested by administration of the questionnaire to 50 caregivers in the same study setting over 2 month period prior to commencement of the study and expert review. Extensive local and international literature survey was done prior to drafting of the questionnaire. Clinical research associates carried out data collection at PDGH and regional hospitals under RDHS. In order to minimize interviewer bias, all clinical research associates were trained by the principal investigator to administer questionnaires. Piloting was carried out in all study settings for 2 months prior to commencement of the study and all data collections were done under direct supervision of the investigators of the study.

Investigators evaluated the association of three factors with acute poisoning in addition to collection of observational data in the prospective study at Anuradhapura Teaching hospital. The three proposed associations were (1) effect of long term medication use by a family member on poisoning with medications, (2) effect of a parent being a farmer and plant poisoning, and (3) effect of a parent being a farmer and pesticide poisoning. This study involved all children in the prospective cohort at Anuradhapura Teaching hospital. A case was defined for the study as a child with acute poisoning following (1) only medicinal poisons, (2) only plant poisons, and (3) only pesticide poisons respectively for the proposed three associations. Controls were defined for each analysis as children in the cohort who did NOT have the particular category of poisoning. Risk factors were defined for the study. Presence of any household member on medication for more than at least 1 month was considered as a risk factor. A parent was considered as a farmer when he or she was engaged in farming as his/her occupation. The controls for the three proposed risk factors were selected within the same cohort and included those with poisoning other than (1) medicinal poisons, (2) plant poisons, and (3) pesticide poisons respectively.

Retrospective observational study was conducted based on Bed head ticket (BHT) data and only limited demography and poison factor related data which could be considered reliable and auditable by discharge registers were collected. Data in the retrospective observational series were collected by the principal investigator himself to minimize record retrieval related bias.

### Data analysis

All data were analysed using SPSS version 19.0. Means and percentages were calculated to present descriptive data in different study settings. The risk factor analysis included children who were in the prospective cohort recruited from Anuradhapura Teaching hospital. The proposed three risk factors were used to create three separate logistic regression models adjusted for age and sex. In these models, each factor was evaluated using univariate analysis for significance levels. Controls were kept as the dependent and each proposed risk factor was submitted as a categorical covariate. Odds ratios were calculated for each risk factor along with 95% confidence intervals (CI).

### Data reliability and auditing

Data collections were subjected to independent audit and close monitoring by South Asian Clinical Toxicology Research Collaboration (SACTRC) and the investigators of the study.

## Results

Total number of children available for analysis was 1621 (ATH prospective series- 383, ATH retrospective series - 625, PDGH prospective series- 371, RDHS prospective series - 242). Table 1 elucidates the variation of demographic factors in different arms of the study.

**Table 1:** Patterns of poisoning characteristics in rural Sri Lanka (ATH - Anuradhapura Teaching Hospital, PDGH – Polonnaruwa District General Hospital, RDHS - Regional Director of Health Services)

Variable	ATH Retrospective series (*N* = 625)	ATH Prospective series(*N* = 383)	PDGH Study (*N* = 371)	RDHS Study (*N* = 242)	Total (*N* = 1621)
1.Male gender	60%	58%	61%	56%	60.0%
2.Age < 5 years	79%	78%	80%	85%	80.0%
3.Unintentional poisoning events	96%	95%	95%	96%	95.6%
4.Mortality	3 (0.5%)	2 (0.5%)	2 (0.5%)	–	7 (0.4%)
5. Transfer rate	55.8%	65%	62.2%	63.3%	60.4%

Among 1621 children who presented with acute poisoning, the majority had been male (956 children) and were under 5 years (1296 children). There were 71 children (4.4%) who had presented with non-accidental poisoning. The majority of intentional poisonings were associated with pesticides (32 children) and plant poisons (27 children). Intentional poisoning with household substances was not observed. Overall transfer rate of children between healthcare centers was 60.4%.

Tables 2 and 3 describes the variation of types of poisons and common poisons in different study settings.

**Table 2:** Patterns of the variation of different types of poisons in different study settings among children with acute poisoning (ATH - Anuradhapura Teaching Hospital, PDGH – Polonnaruwa District General Hospital, RDHS - Regional Director of Health Services)

Poison Category	ATH Retrospective series (*n* = 625)	ATH Prospective series (*n* = 383)	PDGH Study (*n* = 371)	RDHS Study (*n* = 242)	Total (*n* = 1621)
1.Household chemicals	162 (25.9%)	120 (31.3%)	98 (26.4%)	109(45%)	489 (30.2%)
2.Medicines	135 (21.6%)	112 (29.3%)	108 (29.1%)	55(22.7%)	410 (25.3%)
3. Plants	165 (26.4%)	65 (17%)	58 (15.6%)	37 (15.3%)	325 (20.1%)
4.Miscellaneous	114 (18.2%)	49 (12.8%)	61 (16.4%)	18 (7.4%)	242 (14.9%)
5.Pesticides	49 (6.6%)	37 (9.7%)	46 (12.4%)	23 (9.5%)	155 (9.6%)

**Table 3:** Patterns of the variation of common poisons in different study settings among children with acute poisoning (ATH - Anuradhapura Teaching Hospital, PDGH – Polonnaruwa District General Hospital, RDHS - Regional Director of Health Services)

Poison	ATH Retrospective series (*n* = 625)	ATH Prospective series (*n* = 383)	PDGH Study (*n* = 371)	RDHS Study (*n* = 242)	Total (*n* = 1621)
1.Kerosene oil	116 (18.6%)	79 (20.6%)	56 (15.1%)	56 (23.1%)	307(18.9%)
2.*Jatropha circus*	81 (13.0%)	22 (5.7%)	24 (6.5%)	16 (6.6%)	143 (6.6%)
3. Paracetomol	48 (7.7%)	39 (10.2%)	27 (7.0%)	22 (9.1%)	136 (8.4%)
4. *Thevetia peruviana*	36 (5.8%)	10 (2.6%)	14 (3.8%)	8 (3.3%)	68 (4.2%)
5.Organophosphate insecticides	10 (1.6%)	17 (4.4%)	24 (6.5%)	12 (5.0%)	63 (3.9%)
6.Mosquito coil	17(2.7%)	15(3.9%)	20(5.4%)	11(4.6%)	63(3.9%)
7. *Abrus precatorius*	27 (4.3%)	15 (3.9%)	11 (3.0%)	7 (2.9%)	60 (3.7%)
All other poisons	290 (46.4%)	186 (48.6%)	195 (52.6%)	110 (45.5%)	781 (48.2%)

Household chemicals were accountable for 489 acute poisonings (30.2%). The most common poison was kerosene oil in all study settings. Medicinal agents lead to 410 poisoning events (25.3%) whilst plant poisoning accounted for 325 incidents of poisoning (20.0%). The most common plant poison was *Jatropha circus* in all study settings. Other common plant poisons included Oleander and *Abrus precatarius*. Pesticides were the least common among all types of poisons (155 overall with 9.6%). The most common medicinal and miscellaneous agents accountable for poisoning were paracetomol and petrol respectively. The most common pesticide accountable for acute poisoning was organophosphate. Poisoning with household chemicals was higher in the RDHS study (45%) as compared to other prospective observational studies and most of the poisonings were secondary to ingestion of kerosene oil which is used for lighting houses and cooking in remote houses.

The majority of children ingested the poison (1582, 97.6%) while inhalation (37 cases) and skin contact (2 cases) were other routes of poisoning.

### Prospective series at Anuradhapura teaching hospital

Total number of children available for analysis was 383. The majority of children belonged to families of which the parents were employed in agricultural sector (26%), defense service (16%) and manual labour (12%). Eighty percent of parents had received at least secondary education. The effect of proposed risk factors was evaluated only at ATH since data were available only for that study setting. The Table 4 describes effect of proposed risk factors.

**Table 4:** Risk Factors for different types of Poisoning, in respective cohorts

Proposed risk factor	Cases	Controls	Odds Ratio	95% CI (OR)		*P* Value
				Low	High	
1. Medication use by a family member and risk for medicinal agent poisoning	57(44.8%)	55 (21.5%)	3.16	2.14	4.28	< 0.001
2. A parent being a farmer and risk of poisoning with pesticides	15(14.2%)	22(7.9%)	1.61	0.94	2.27	0.068
3. A parent being a farmer and risk of poisoning with plants	22 (20.7%)	43(15.5%)	1.19	0.80	1.57	0.220

The analysis revealed that whilst medication use by a family member was associated with significantly high risk of poisoning with medicinal agents in children, there was no significantly high risk of either pesticide or plant poisoning among children of farming parents as compared to non-farming parents.

Eighty five poisoning events (22%) occurred in the kitchen area and the most common substance to be poisoned in kitchen area was kerosene oil (74/85 cases, 87%). Altogether home and home garden were the location for poisoning in 304 incidents (79.4% of acute poisoning events).

First aid measures were practiced on 113 children (29.5%) by their care givers following recognition of the poisoning event. There were four events of aspiration pneumonia secondary to forceful serving of coconut milk after ingestion of kerosene oil. Two children who were given plenty of water following organophosphate ingestion developed pneumonia following aspiration. Care takers were unaware of harmful effects in all cases. Table 5 describes the first aid measures offered to children by their parents following the poisoning event, the duration of delays and reasons for delayed presentation of children with acute poisoning.

**Table 5:** Type of “First aid” practices, the duration of delay and reasons for delayed presentation of children with acute poisoning

First aid practice	Number of children	Percentage (%)
1.Serving water	53	13.8
2.Serving coconut milk	34	8.9
3.Serving milk	8	2.1
4.Finger insertion to throat	8	2.1
5.Serving soap water	4	1.0
6.Serving lime water	3	0.8
7.Thumping over back to assist spitting of the poison	2	0.5
8.Offering mashed Nutmeg	1	0.3
Duration of delay		
Less than 30 min	119	31.1
30–60 min	145	37.8
1–2 h	53	13.8
2–6 h	29	7.6
More than 6 h	37	9.9
Reasons for delayed presentation		
1. Lack of concern regarding urgency of the situation	65	16.9
2. Lack of knowledge regarding possible complications	64	16.7
3. Lack of transport facilities in emergencies	52	13.5
4. Lack of financial resources	32	8.3
5. Child had not told about incident until symptoms occur	11	2.8
6. Delayed attention by the medical team	1	0.3

149 children (38.9%) remained asymptomatic following ingestion of the poison. Among those who became symptomatic (*n* = 234: 61.1%), 73 (31.2%) children developed immediate onset symptoms. Forty seven children (47/234, 20.1%) had symptoms within 30 min and 66 children (28.2%) became symptomatic within 30 min to 1 hour. Fifty children (50/234, 21.4%) had delayed onset symptoms at least after 1 hour. The most common reasons for delayed presentation at emergency center had been lack of concern by family members regarding the urgency of the situation (16.9%) and lack of knowledge regarding possible complications (16.7%).

### Management of complications following acute poisoning

Complications were reported in 12.5% of children with acute poisoning. Sixty five children (17%) were offered gastric decontamination at peripheral hospitals whilst 97 children (25.4%) were offered gastric decontamination at the teaching hospital. Seven children (1.8%) needed admission to the paediatric intensive care unit. Antidotes were required in 20 children and the most commonly used antidote was N- Acetyl Cysteine for paracetomol intoxication. Formal psychological review was arranged only in three children following referral to the consultant psychiatrist. Reported complications are described in Table 6.

**Table 6:** Complications following acute poisoning

Complication	Number	Percentage
1.Chemical Pneumonitis and aspiration pneumonia	20	5.2
2.Acute hepatic injury	10	2.6
3.Cardiac arrhythmias	5	1.3
4.Acute dystonic reactions	3	0.8
5.Convulsions	2	0.8

### Mortality

Seven deaths were observed during the 7 year period of study. One child died following severe aspiration pneumonia secondary to kerosene oil ingestion and harmful emesis induction measures given by care givers. Four children succumbed to fatal cardiac arrhythmias related to Oleander poisoning. Two children died following fatal organophosphate poisoning. The case fatality rates associated with Oleander, organophosphate pesticides and kerosene oil were 5.9%, 3.2% and 0.3% respectively.

## Discussion

We observed that most children who ingested poisons were less than 5 years and it is consistent with studies published from Asia [12]. Younger children were poisoned more commonly with household chemicals as compared to older children who ingested medical and plant poisons at a higher percentage and similar observations were made in other studies from Asia [13]. Different poisoning patterns were observed in studies from Africa [14], Middle East [15], Australia [16], Europe [17] and North America [18] where medicines were identified as the most common type of poisons.

The current study reported that a family member being on long term medication was associated with a significantly higher risk for poisoning by a medicinal toxin in the child. Children may observe other family members taking medicines, and the development of imitation behaviour around the age of 2 years may partially explain the higher risk for medicinal poisoning in young children [19]. Previous history of poisoning was also observed to have a significantly high risk for further acute poisoning in children [20]. Children can be exposed to plant toxins in agricultural fields [21] However, the current study failed to observe any significant association between parents being farmers and plant poisoning. Long term hazards of pesticide exposure at home on children are well recognized [22]; however, the current study could not identify any association between parents being farmers and acute pesticide poisoning in children.

A previous Sri Lankan study concluded that male children were more affected than female children with a risk ratio of 3:2 [8]. The current study based on children from rural Sri Lanka observed similar risk ratios in all study settings and the observations were consistent with similar international studies [23]. Male children outnumbered female children among all age groups examined up to 12 years of age in the current study and similar observations have been made in studies from India [24], Pakistan [25], USA [26], Europe [27], and Australia [28].

Pesticides were the least common type of poison as compared to household chemicals, plant poisons and medications. This could be due to the fact that parents were more cautioned in storage of pesticides compared to other poisons types, which most parents would not anticipate that children would ingest or may have the potential to cause complications. Proportions of other types of poisons vary markedly in all studies and observations were inconsistent. This could be explained as the effect possible confounders in different locations such as care takers taking long term medications, access to poisonous plants, and poor storage. The majority of parents did farming and manual labor as a living and similar observations have been noted in studies from rural South Asia [29].

Home and home garden were the location for more than 80% of acute poisoning events and it was similar in other studies from South Asia (85.7%) [30] and Central Asia (89%) [31]. Bed room area of the house was the most common location for poisoning in studies from developed countries where medicinal agents were implicated as the most common type of poisons [32]. Most Asian studies found kerosene oil as the most common poison in the paediatric age group whilst most of those poisoning events occurred in kitchen area [30, 31].

We observed that 69.1% of children were brought to primary care hospital/emergency unit within 1 hour of poison ingestion by their parents. It is a much shorter time compared to other studies in South Asia that reported 60% casualties within 3 h and 77% in 6 h [33]. Delayed presentation to emergency unit following acute poisoning is associated with increased risk of complications [34]. Child remaining asymptomatic after ingestion, lack of identity of the ingested substance as a poison, and small ingested amount of poison had kept a higher threshold for some parents to seek urgent medical attention. Lack of transport facilities in more rural territories of NCP is reportedly a barrier for timely medical management [35] and 13.5% of parents in the current study had transportation difficulties in reaching the primary healthcare facility. Public transport systems hardly exist during night time in these regions whilst many dwellers remain at their home premises at nighttime owing to the fear of wild elephants. Ignorance, and financial and transport difficulties have been reported as reasons for delayed presentations in similar studies from South Asia [36].

The percentage of decontamination was higher compared to studies in developed countries [37]. Possible reasons for this observation includes higher occurrence of plant and pesticide poisonings in the current study. As the percentage of young children was higher in the current study, the amount of poison ingested in many instances was uncertain and clinicians had to offer treatment based on parents’ history. However, most children did not need any active intervention in the current study, and the finding is comparable with other studies in literature [38].

A previous Sri Lankan study had found that medicines accounted for 32% of all acute poisonings in the paediatric age group [38]. We observed a lesser contribution from medicines for acute poisoning in children (range 21.6–29.2%) and mortality was zero. We observed a higher percentage of deliberate ingestions of plants compared to previously published, urban Sri Lankan studies [39] and the types of plants observed were different.

Coconut milk as a first aid measure to induce emesis following kerosene oil poisoning had been substantially low in our study (26.6% vs. 77%) compared other Sri Lankan studies [6] and it likely indicates better education and awareness among current generations as compared to previous generations. Also there had been a substantially lower occurrence of pneumonitis following kerosene oil poisoning in our study (20.2% vs. 57%) compared to previous Sri Lankan studies [6].

Studies from other countries in South Asia have reported higher percentages (18.1%) of pesticide poisoning [40] compared to our study (9.4%). Increased awareness among farmers regarding poisoning risks and strengthening of legislations for controlling unwarranted use of pesticides are likely reasons for this difference and recent decrease in the number of pesticide poisoning cases in Sri Lanka. This figure in rural Sri Lanka was higher compared to what was seen in studies involving more urban populations in Sri Lanka [39].

The most common symptoms in children with acute poisoning were neurological in several studies from West Asia [41] and Europe [42] and were mostly following medication poisoning. We observed that combined gastrointestinal symptoms as the most common clinical manifestation followed by respiratory and neurological symptoms. Complications were observed in 6% of children with acute poisoning in a study from Pakistan [43] and the most common complication was chemical pneumonitis (4%). We observed an overall complication rate of 12.5% with 5.2% resulting from aspiration/chemical pneumonia mostly secondarily to kerosene oil ingestion. As an overview, the findings show mostly similar poisoning patterns compared with countries in South Asian region and different poisoning patterns compared with other geographic regions in the world.

Current study revealed that 979 children (60.4%) were transferred from primary care hospital to secondary/tertiary care hospitals for further management in spite of the majority of poisoning events being not associated with any medical complications. Transferring of patients between hospitals is costly [7] given the limited availability of resources in rural Sri Lankan hospitals. Senarathna et al. studied social dynamics in rural Sri Lankan hospitals based on patients with acute poisoning and appreciated that healthcare workers in peripheral hospitals as being more interactive, receptive and having more positive attitudes towards managing patients with poisoning [44]. It is valuable in this background to educate them on the nature and outcomes of poisoning among children. Empowerment of health staff would likely limit transfers and expenditure on management of these children.

## Conclusion

Children with acute poisoning in rural Sri Lanka were predominantly preschoolers, and male children were at a higher risk. They are poisonined mostly within their own housing premises. Kerosene oil, in addition to being the most common poison, had additional risks of aspiration pneumonia secondary to potentially hazadrous first aid measures practised by the care givers. Long term medication use by family members was associated with a significantly high risk of poisoning with a medicinal agent in children. Complications though rare are potentially preventable through community education and awareness on timely attention to seek medical care and avoidance of potentially harmful first aid practices.

It is equally important that all child health care providers are well educated regarding initial response to symptomatic children after poisoning, subsequent triage and supportive care for more efficient utilization of available limited resources.

## Additional file

Additional file 1: Multi-structured data collection questionnaire. (PDF 137 kb)
