# Supplementary material for: Patterns and outcome of acute poisoning among children in rural Sri Lanka
Source: BMC Pediatr. 2018 Aug 18;18:274. doi: 10.1186/s12887-018-1246-0 (PMC6098835; doi:10.1186/s12887-018-1246-0)
Supplement: Supplementary file 1 — Multi-structured data collection questionnaire. (PDF 137 kb) [file 12887_2018_1246_MOESM1_ESM.pdf]

Patterns and outcome of acute poisoning among children in rural Sri Lanka - study questionnaire

Data of data collection: ……/…../…….	Hospital: ……………………………
Date of admission: .…/……/…….	Ward: …………

Part 1

1. Basic demographic data

1. Name: ……………………………………………………………
2. BHT Number: ………………………..
3. Age: ……………….
4. Gender: male / female
5. Residential address: ……………………………………………………………………
6. Medical officer of health (MOH) division: ……………………………………..
7. Public health midwifery (PHM) division: ……………………………………….
8. Divisional secretariat: ………………………………………
9. Parents education level: Father: ……………………….. Mother: ………………………..
10. Parents’ profession / occupation: Father: ……………………. Mother: …………………
11. Whether at least one of the members in household is on long term medication: Yes/ No
12. Previous history of poisoning : Yes/ No
13. Ethnicity: ……………………….. Religion: ……………………………
14. Location of the poisoning event – Home garden/ Kitchen/ Bedroom/ Living room/ Other – please specify: ……………………

Part 2

2. Nature of poisoning: Deliberate / Accidental

If deliberate, the precipitating event:
…………………………………………………………………………………………………
…………………………………………………………………………………………………

If accidental, the precipitating event:
…………………………………………………………………………………………………
…………………………………………………………………………………………………

3.1. Type of poison: Household poisons/Medicines/ Poisonous plants/ Pesticides/ Miscellaneous

3.2. General/trade name of the poison: ……………………………………………

3.3. Chemical/ Scientific name of the poison (If available):
………………………………………………..

3.4. Method of poisoning – Ingestion/ Inhalation/ Skin contamination/ Other please specify :
……………………………………………….

4. Quantity of poison: ………………………………………………….

5.1. First aid measures taken by the care takers

1. Coconut milk to induce emesis Yes/No
2. Milk to induce emesis Yes/No
3. Water to induce emesis Yes/No
4. Soap water to induce emesis Yes/No
5. Specify, if any other
   ………………………………………………………………………………………………

5.2. Their perceived benefit from the intervention of interest:
……………………………………………………………………………………………
5.3. Any detrimental effects of home remedies:
……………………………………………………………………………………………

6.1. Symptoms before presenting to hospital

Cough / Shortness of breath / Hiccups / Giddiness / Abdominal pain / Unconsciousness / Sore throat / Palpitations / Drowsiness/Vomiting/ Loose stools/ Dizziness

Specify if any other;
………………………………………………………………………………………………

6.2. Time for onset of symptoms following poisoning: ……..hrs ……….min.

6.3. Time for presentation to pediatric emergency unit: ………..hrs ……….min.

6.4. What are the possible reasons for delayed presentation?
1. Lack of transport in emergencies Yes/ No
2. Lack of financial resources Yes/ No
3. Lack of knowledge regarding possible complications Yes/ No
4. Lack of concern regarding the urgency of the situation / need for intervention Yes/ No
5. Delayed attention by the emergency medical team Yes/ No
6. Any other reason
………………………………………………………………………………………………

6.5. Transferred patients:
1. Name of the primary hospital: …………………………

2. Medical induction of emesis: Yes/No
3. Reason for transfer:
…………………………………………………………………………………………………

7. Short history from admission to discharge / transfer/death:

1. Medical induction of emesis Yes/No
……………………………………………………………………………………………………
2. Observation for development of complications Yes/No
3. Emergency management drugs/ procedures Yes/No
……………………………………………………………………………………………………
4. ICU admissions and management at ICU Yes/No
……………………………………………………………………………………………………

5. Prescription of antidotes/ special medicines Yes/No
……………………………………………………………………………………………………
6. Management of poisoning related complications Yes/No

……………………………………………………………………………………………………
7. Psychological intervention/ Psychiatric referral Yes/No
……………………………………………………………………………………………………
8. Family intervention/ Family counseling Yes/No
……………………………………………………………………………………………………
9. Details about medical investigations -CXR / Blood toxin levels / Serum electrolytes / Renal function test / Liver function test / Specify if any other
……………………………………………………………………………………………………
……………………………………………………………………………………………………
……………………………………………………………………………………………………
……………………………………………………………………………………………………
………………………………
10. Transfer to other hospitals: Where to: ……………………………………………..

<u>Part 3</u>

8. Outcomes of poisoning

1. Chemical pneumonitis or aspiration pneumonia yes/no
2. Pnuemothorax / Pneumopericardium/ Pneumomediatinum yes/no
3. Acute renal injury yes/no
4. Acute liver injury yes/no
5. Cardiac arrhythmias yes/no
6. Hypotension yes/no
7. Convulsions yes/no
8. Death of the child yes/no
9. Specify if any other - ……………………………………….
10. No complications
